# Synaptic dynamics contribute to long-term single neuron response fluctuations

**DOI:** 10.3389/fncir.2014.00071

**Published:** 2014-07-01

**Authors:** Sebastian Reinartz, Istvan Biro, Asaf Gal, Michele Giugliano, Shimon Marom

**Affiliations:** ^1^Network Biology Research Laboratories, Faculty of Electrical Engineering, Technion - Israel Institute of TechnologyHaifa, Israel; ^2^Department of Physiology, Faculty of Medicine, Technion - Israel Institute of TechnologyHaifa, Israel; ^3^Theoretical Neurobiology and Neuroengineering Lab, Department of Biomedical Sciences, University of AntwerpWilrijk, Belgium; ^4^Department of Computer Science, University of SheffieldSheffield, UK; ^5^Brain Mind Institute, Swiss Federal Institute of Technology of LausanneLausanne, Switzerland

**Keywords:** single neuron, synaptic dynamics, response fluctuations, electrical stimulation, cortical culture, microelectrode array, cortical slice, patch clamp

## Abstract

Firing rate variability at the single neuron level is characterized by long-memory processes and complex statistics over a wide range of time scales (from milliseconds up to several hours). Here, we focus on the contribution of non-stationary efficacy of the ensemble of synapses–activated in response to a given stimulus–on single neuron response variability. We present and validate a method tailored for controlled and specific long-term activation of a single cortical neuron *in vitro* via synaptic or antidromic stimulation, enabling a clear separation between two determinants of neuronal response variability: membrane excitability dynamics vs. synaptic dynamics. Applying this method we show that, within the range of physiological activation frequencies, the synaptic ensemble of a given neuron is a key contributor to the neuronal response variability, long-memory processes and complex statistics observed over extended time scales. Synaptic transmission dynamics impact on response variability in stimulation rates that are substantially lower compared to stimulation rates that drive excitability resources to fluctuate. Implications to network embedded neurons are discussed.

## 1. Introduction

Ongoing single neuron electrical activity shows significant fluctuations over extended time scales (from milliseconds up to several hours). Whenever data recording is sufficiently prolonged to enable a proper analysis of extended time scales, complex statistics of the spike time series emerges. They typically exhibit signatures of what statisticians define as “long-memory processes,” reflected in long range temporal correlations and seemingly unbounded spectral density at low frequencies (Baillie, 1996). These complex statistics are present whether the system is spontaneously active, when activated via natural sensory modalities, or when local electrical stimulation is delivered *in vivo* as well as *in vitro* (Teich et al., 1990, 1997; Heck et al., 1993; Carandini, 2004; Mazzoni et al., 2007).

Two possible sources for temporally complex single neuron activity have been discussed in the literature. The *first* is the inherently non-stationary activity of recurrent neural networks (Segev et al., 2002; Beggs and Plenz, 2003; Pasquale et al., 2008); the firing of individual neurons reflect the statistics of the population that they are embedded in (Arieli et al., 1996; Azouz and Gray, 1999; Kisley and Gerstein, 1999). The *second* source of complexity arises from kinetics of ionic channels underlying excitability at the single neuron level; over extended time scales these kinetics are dominated by long-lasting states, giving rise to complex firing statistics and long-memory processes (Toib et al., 1998; Marom, 2009; Gal et al., 2010; Gal and Marom, 2013, 2014).

But there is one more potential source for single neuron response fluctuations over extended time scales: Under physiological conditions, the cell response is elicited by presynaptic neurons through a synaptic population. It has been reported that a relatively stable subset of synapses collectively becomes active and releases neurotransmitter, either in response to a unique stimulus or as part of ongoing network activity (Jia et al., 2010; Chen et al., 2013). This stimulus specific ensemble of synapses constitutes an interface between the network and a given cell and might significantly modulate the statistical structure of network input to the cell. Moreover, the dynamics of synaptic transmission might itself be complex (e.g., Lowen et al., 1997; Varela et al., 1997) and thus in itself become a potential source of long-memory processes and complex statistics of neuronal activity. Here, we study the contribution of synaptic dynamics to the temporal complexity of the neuronal response over extended time scales. To this aim, we have developed means to activate single neurons *in vitro* through a synaptic population, while suppressing ongoing activity from the surrounding network. We have identified measures that enable a clear separation between the two determinants of neuronal response variability under these conditions, that is: the dynamics of membrane excitability and the dynamics of synaptic transmission (including pre-/post-synaptic and dendritic mechanisms). We show that within a range of stimulation frequencies (2–6 Hz) that is similar to the measured firing rate of cortical neurons *in vivo* (Abeles, 1991), the changing state of this synaptic ensemble is a key determinant of long-term temporal statistics of neuronal response.

## 2. Materials and methods

### 2.1. Experimental preparations

Cortical tissue was obtained from newborn rats (Sprague-Dawley) within 24 h after birth, mechanically and enzymatically dissociated following standard procedures (Marom and Shahaf, 2002). Approximately 1.3 × 10^6^ cells were seeded on poly-ethylene-immine (0.01% in 0.1M Borate buffer solution) pre-treated substrate-integrated microelectrode arrays (MEAs). Before usage, cultures were allowed to develop mature networks over a time period of 2–4 weeks. Cultures were bathed in Eagle's Minimum Essential Medium (MEM), supplemented with heat-inactivated horse serum (5%), glutamine (0.5 mM), glucose (20 mM), and gentamycin (10 μg/ml), and maintained in an atmosphere of 37 °C, 5% *CO*_2_, and 95% air during incubation as well as during recordings.

In a subset of the experiments, parasagittal brain slices (300 μm thick) were acutely prepared in ice-cold extracellular solution, from the rat somatosensory cortex (postnatal 14–21 days), as in Koendgen et al. (2008). Slices were incubated at 35°C for 60 min, before being mounted over glass-substrate arrays of 3D tip-shaped Pt microelectrodes (Qwane Biosciences, Switzerland). 3D-MEAs were previously coated with cellulose nitrate (Protran, Fisher Scientific, Belgium; 0.14 mg/ml in 100% Methanol), and replaced the bottom of an upright microscope chamber (Scientifica, UK). The protocols were approved by the Inspection Committee on the Constitution of Animal Experimentation at the Technion (no. IL-099-08-10) and by the local Ethical Committee of the University of Antwerpen.

### 2.2. Pharmacological manipulations

**Figure 1:**
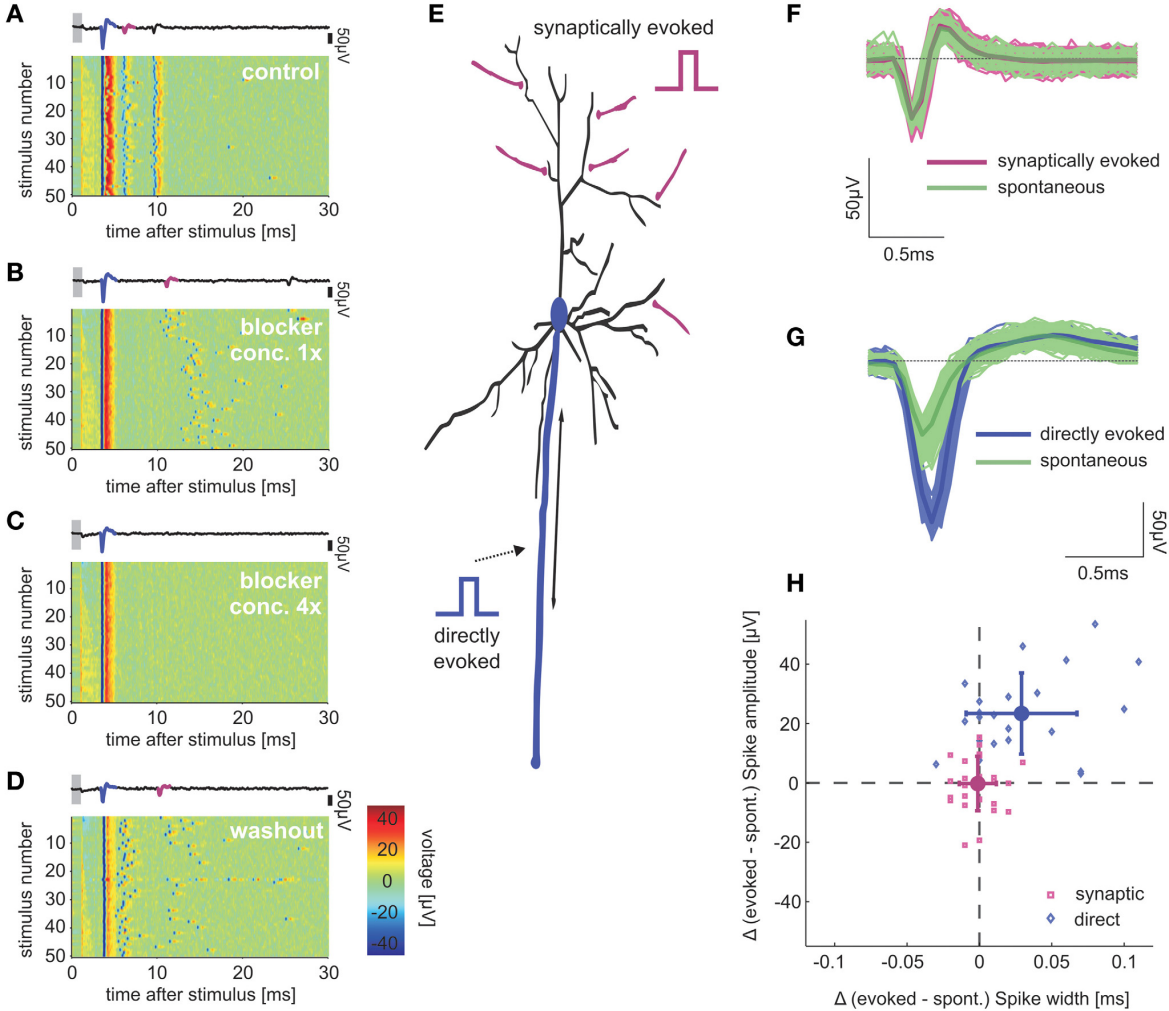
**Direct and synaptically mediated response spikes**. Pseudo-color plots **(A–D)** show voltage traces detected by an electrode that records both directly evoked and synaptically mediated spikes, in different concentrations of synaptic blockers: **(A)** Control solution with no blockers; **(B)** Added blockers (8 μM APV, 4 μM CNQX, 2 μM BIC); **(C)** Four-fold concentrations compared to **(B)**; and **(D)** Back to control solution after washing out the blockers. Single voltage traces above each panel depict direct (blue) and synaptic (magenta) mediated spikes in the first trace of each condition. Focusing on the immediate synaptically evoked responses, we did not consider secondary spikes of the synaptically activated neuron. Gray bars mark blanking period. Color bar in **(D)** indicates voltage-color correspondence, identical in **(A–D)**. **(E)** Conceptual scheme of the two local responses to direct (blue) and synaptic (magenta) activation. Sketches of stimulation signal illustrate potential locations of stimulation electrode for each of the two response types. **(F)** Shapes of spikes generated spontaneously by the network (green) or by synaptic stimulation (magenta); average traces are depicted with bold lines. **(G)** Shapes of spikes of another neuron that was directly stimulated (blue) and activated by the network (green). **(H)** Synaptic mediated response spike waveforms resemble spontaneous network-mediated spike shapes. Each dot represents the difference in mean amplitudes and half-widths, between evoked and spontaneous spikes in one single neuron. Mean and σ of the distributions are depicted (big markers, errorbars). Spike half-width was determined as the width at half maximum amplitude, using an extrapolation between the two points closest to the half maximum amplitude.

Network spikes are events of synchronous burstings that spread and reverberate throughout the network which can occur spontaneously as well as in response to electrical field stimulation (Eytan and Marom, 2006). Hence, they might interfere with our capacity to interpret the impacts of stimulation on response dynamics (Wallach and Marom, 2012; Weihberger et al., 2013). Network spikes may be suppressed by pharmacological blockage of NMDA channels in cultured cortical networks (Robinson et al., 1993; Jimbo et al., 2000; Bonzano et al., 2006). To enhance our ability to relate between stimuli and evoked responses, network spikes have been suppressed by bath applying 60–120 μM D-2-Amino-5-phosphonovaleric acid (APV) (see also Bonifazi et al., 2005). Main excitatory (AMPA, NMDA) and inhibitory (GABAA) synaptic receptors may be blocked in order to suppress synaptic transmission in cultured cortical networks (Gal et al., 2010). The addition of 6-cyano-7-nitroquinoxaline-2,3-dione (CNQX), APV, and bicuculline methiodide (BIC) to the bathing solution can be applied to suppress synaptic activity (Figure 1). Further experimental manipulations showed that the AMPA receptor is the prime mediator of these immediate synaptically evoked spikes, as they are insensitive to APV and BIC; furthermore, it is sufficient to apply 10–20 μM CNQX to abolish them completely (not shown). The latter was previously shown to block excitatory synaptic transmission between synaptically coupled neurons (Bi and Poo, 1998). Thus CNQX (10–20 μM) was added, when the synaptic mediation of early neuronal responses needed to be confirmed, following stimulation experiments. In all cases of pharmacological manipulations, 30 min were allowed for their effects to take place before experiments were resumed. All the chemicals were from Sigma-Aldrich or Merck.

### 2.3. Extracellular recording and stimulation

Arrays of 60 Ti/Au/TiN extracellular electrodes with electrode spacing/diameter 500/30 μm, were used [Multi Channel Systems (MCS), Reutlingen, Germany]. A commercial amplifier (MEA-1060-inv-BC, MCS) with frequency limits of 150–3000 Hz and a gain of ×1024 was used to reduce noise. Data was digitized with an acquisition board (PD2-MF-64-3M/12H, UEI, Walpole, MA, USA) and sampled at a frequency of 16 Ksample/s per channel. Monophasic, 200 μs square pulse 100–1000mV voltage stimulation through extracellular electrodes was performed (Wagenaar et al., 2004), using a dedicated stimulus generator (STG 1004, MCS). Data pre-processing and online event detection were performed using a Simulink-based (The Mathworks, Natick, MA, USA) xPC target application (see Zrenner et al., 2010 for details). Extracellular spikes were detected online by threshold crossing (8 × STD) of the raw voltages. Spike times and shapes, as well as −10 to +50 ms voltage traces triggered by each stimulus, were recorded from all electrodes. When we state the number of used networks, this is congruent with the number of experiments. The data were analyzed by custom MATLAB scripts (The Mathworks, Natick, MA, USA).

### 2.4. Intracellular recording and stimulation

In slice experiments, large layer 5 (L5), regular-firing pyramidal cells (McCormick et al., 1985) were visualized (40×) by differential interference contrast IR-microscopy and whole-cell patch-clamp recordings made at 32°C from the cells soma. The extracellular solution, containing (in mM): 125 NaCl, 25 NaHCO3, 2.5 KCl, 1.25 NaH2PO4, 2 CaCl2, 1 MgCl2, 25 glucose, bubbled with 95% O2, 5% CO2, was perfused at a minimal rate of 1mL/min, while the intracellular solution contained (in mM): 115 K-gluconate, 20 KCl, 10 4-(2-hydroxyethyl)-1- piperazineethanesulfonic acid (HEPES), 4 adenosine triphosphate-Mg, 0.3 Na2-guanosine triphosphate, 10 Na2-phosphocreatine, pH adjusted to 7.3 with KOH. Neighboring pairs of 3D-MEAs electrodes, located in correspondence of L1-L2/3 or L5-6, less than 1100 μm from the cell apical dendrite, were selected to deliver extracellular biphasic current pulses (symmetric, positive step first, 200 μs) by a stimulus generator (STG4000, MCS), with 3–5 s of recovery interval. Synaptically evoked action potentials (APs) were recorded intracellularly, upon 3D-MEA repeated stimulation in L1-L2/3 (≥500 μm from the cell soma). Directly evoked APs were instead recorded in the same neurons following bath application of APV, CNQX, and SR-95531 (GABAzine) (50, 10, and 10 μM, respectively), and upon 3D-MEA repeated stimulation in L5-6, in proximity of the cell soma. In order to favor the stability of the recordings, stimulus amplitudes were selected interactively (i.e., repetitions, range, resolution), cell-by-cell, and shuffled to reduce the adverse impact of drifting input resistance. For comparing results across cells, counteracting small drifts in the resting membrane potential (*V*_rest_), and slightly depolarizing neurons to *in vivo*-like potentials, a real-time proportional-integral (PI) feedback controller (*P* = 10 pA/mV, I = 100 pA/(*mV* × *s*)^−1^; was implemented in Simulink-xPC and employed. This enforced *V*_rest_ at the same value, 3–6 mV below the neuron's threshold, upon continuous injection of small (76 ± 64 pA) intracellular currents. The controller was transiently disabled, immediately before the delivery of each extracellular stimulus by holding the last injected current value, and enabled again, 500 ms after.

### 2.5. Time series analyses

Analyses of rate statistics were performed as in Gal et al. (2010). For each experiment, the neuron spike train was represented as a single point-process (i.e., without reference to stimulation time). Periodograms (Scharf et al., 1995) and Fano Factor analyses (Lowen and Teich, 1996) were used to estimate the complexity of the resulting time series. The periodogram, an empirical estimator for the power spectral density of a process, was computed over the count sequence of the spike train with bin size of 1 s. For Fano Factor analysis, a count sequence was calculated using logarithmically spaced bin sizes. For each count sequence, the Fano Factor (variance to mean ratio) was plotted as a function of the bin size. In both measures, the scaling exponents were estimated by linear regression of the logarithm of the data. Long-term spike latency and amplitude statistics were performed on averaged values. Welch's averaged modified periodogram method (Welch, 1967) was used to estimate the power spectral density. The scaling exponents were estimated as described above. First half hour of stimulation was excluded in order to avoid transient effects.

## 3. Results

The results are presented in two sections. The first concerns validation of a method that caters to long-term measurements of synaptically evoked spikes. The second section describes the use of this method in characterization of long-term fluctuations of synaptically evoked responses at the single neuron level, estimating the contribution of synaptic dynamics to these fluctuations.

### 3.1. A method to estimate the impact of synaptic transmission on spike generation over extended time scales

As pointed out by Wagenaar et al. (2004) and Bonifazi et al. (2005), two types of spikes are evoked within the first 20 ms following field electrical stimulation of *in vitro* cortical neurons, using substrate-integrated arrays of microelectrodes (MEAs). These two types differ in their origin: (1) spikes *directly evoked* by depolarization of the somatic or axonal cellular membranes, which are by definition insensitive to synaptic blockers. Especially axon initial segments or other accessible axon positions have been described as sites of direct neuronal activation, which can therefore be also termed antidromic activation (Tehovnik et al., 2006; Histed et al., 2009). (2) spikes whose occurrence is sensitive to synaptic blockers, termed *synaptically evoked spikes*. These two types of spikes are illustrated in Figures 1A–D, plotting single electrode recordings while systematically changing the synaptic blockers concentrations. As panel A shows, both types of spikes appear reliable and temporally precise. In contrast to directly evoked spikes, the latency and temporal variation of synaptically evoked spikes increase with blocker concentrations (Figure 1B). Figures 1C,D shows that synaptically evoked spikes are completely but reversibly blocked by 32 μM APV, 16 μM CNQX and 8 μM BIC.

Direct activation of a single neuron, either by intra-cellular, extra-cellular or optogenetic stimulation is a widely used and valuable experimental technique. But in physiological contexts, activation of neurons occurs by populations of synapses rather than by immediate exposure of voltage-gated ion channels to sudden changes in electric fields. The dynamical characteristics of directly evoked spikes might be quite different from those of spikes that are triggered by network activity under natural conditions. Are synaptically evoked spikes, such as those demonstrated in Figures 1A,B,D, elicited using the method described above, more similar to naturally evoked spikes? To answer this question we took advantage of the fact that our experimental setup enables access to spontaneously occurring synaptically mediated spikes: these spikes are evoked by the ongoing, spontaneous network activity, and may be compared to the acclaimed synaptically evoked spikes that we generate by stimulating synaptic input pathways. Our comparative analysis focused on the spike shape as it is sensitive to past activity, mediated by dynamics of threshold and resting potential (e.g., Henze and Buzsaki, 2001; de Polavieja et al., 2005). Moreover, variations in spike shapes are believed to be physiologically functional (Shu et al., 2006; Boudkkazi et al., 2011). We have used recordings (≥1 h) of spontaneous activity preceding each stimulation session, and as seen in Figures 1F–H, the waveforms of synaptically evoked spikes and spontaneously occurring spikes are practically identical, while markedly differing from waveforms of directly evoked spikes. Figure 1H summarizes (*n* = 12 networks) these differences in waveform between spontaneous, synaptically (*n* = 22 neurons) and directly (*n* = 27 neurons) evoked spikes (*t*-test, *p* < 0.001 for differences in spike amplitudes and widths), after verification that results are free of temporal trends. Spike waveforms of individual neurons change within bursts (e.g., Gray et al., 1995), therefore exclusively spikes with >50 ms previous quiescent period were considered in spontaneous data.

**Figure 2:**
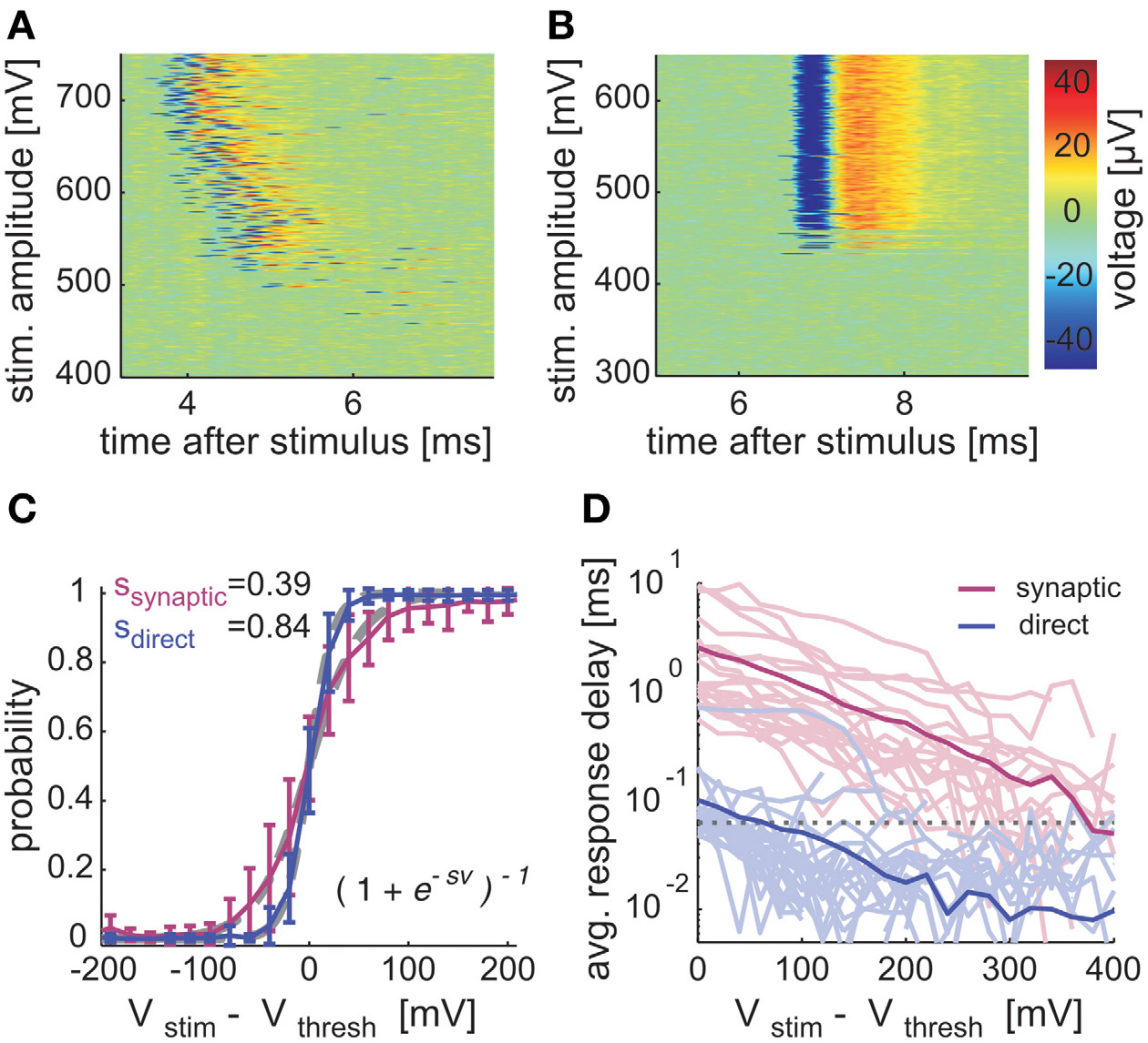
**Input-output and latency in synaptically and directly evoked spikes**. Voltage traces of synaptic **(A)** and direct **(B)** responses sorted by stimulation amplitude. Stimulation amplitudes were delivered (at 0.25 Hz) in 600 steps between 200 and 800 mV in randomized order (zoom into dynamic range). **(C)** Averaged I/O relations, aligned to threshold stimulation amplitude. Logistic functions (gray dashed lines) were fitted to the averaged data. **(D)** Deviation of response latencies from threshold to V_100%_, plotted on a semi-logarithmic scale. Bold lines indicate population mean values; dashed line marks the temporal resolution of the recording setup. In **(C,D)**, networks (*n* = 12, 49 different neurons) were stimulated (≤0.333 Hz) with randomized amplitudes between 200 and 800 mV; fast synaptic transmission was blocked subsequently with ≥10 μM CNQX. Blocked and unblocked responses were accordingly classified as synaptic mediated (*n* = 21) and directly evoked (*n* = 28) responses. Electrodes, recording responses that could be clearly attributed to a single neuron by spike shape and amplitude and spanned the full response range (from 0 to 1 probability) in the applied stimulation amplitudes, were used. Responses with spike delays beyond 20 ms latency at 800 mV were excluded.

Beyond shape and sensitivity to pharmacology, synaptically evoked spikes differ from directly evoked spikes also in their sensitivity to stimulation amplitudes, their input-output (I/O) relations. The absolute amplitude of stimulation that is required in order to evoke a spike, direct and synaptically, reflects a combination of experimental factors that are foreign to the present subject matter; these include, for instance, the distance between the stimulating electrode and the neuron, as well as the spatial configuration of the neuronal arborization. We define threshold stimulation amplitude as the stimulation voltage needed in order to elicit a response probability of 0.5. The threshold stimulation amplitudes in synaptically and directly evoked spikes are 399 mV (±84, mean ± *SD*; *n* = 21 neurons) and 485 mV (±125, mean ± *SD*; *n* = 28 neurons), respectively. Figures 2A,B shows examples of voltage traces, evoked by repeated stimuli with different amplitudes applied in random order, sorted for purposes of presentation. In panel A, the response characteristics of a synaptically stimulated neuron are shown. Panel B shows responses evoked by direct stimulation. Unlike the near stepwise change of the direct stimulation regime between “no-response” mode to a reliable 1:1 response mode, the synaptically stimulated neuron exhibits a gradual increase in response probability with increasing stimulation amplitude. The statistics of this observation (for more than 20 neurons of each group) are shown in Figure 2C. In direct responses, the I/O slopes are two-fold steeper compared to synaptic responses.

Note, in Figures 2A,B, the existence of two temporal observables that characterize evoked responses: the *latency* and the *delay*. The first is defined as the time between stimulation and peak of the elicited spike. It depends on experimentally uncontrolled spatial relations between stimulation electrodes and the neuron, and distributes similarly in neurons of both experimental conditions (7 ± 3.6 ms, mean ± *SD*; *n* = 28 and 6.22 ± 2.44 ms, mean ± *SD*; *n* = 21 for directly and synaptically activated neurons, respectively). The second observable, the response *delay*, is defined as the actual observed latency in a given trial subtracted by the shortest latency evoked in a given neuron. As seen in Figure 2A, spike delays may change as a function of the stimulation amplitude. Figure 2D summarizes the spike delays dependency on the stimulation amplitude, across many experiments. While direct and synaptically evoked spike delays increase with decreasing amplitudes, the range of synaptically evoked spike delays is significantly wider. In fact, the minor delays of direct response latencies at threshold stimulation amplitude (i.e., see Figure 2D x-axis *V*_stim_−*V*_thresh_ = 0) barely exceed the temporal resolution of our recording system.

In this context we have also analyzed the distribution of distances between stimulation and recording electrodes in the two modes of stimulation (direct and synaptic). In synaptic responses, the majority of stimulating-to-recording electrode pairs are neighbors (median = 0.71 mm), in contrast to directly evoked responses, where neighboring electrodes occur in less than 20% of the cases (median = 1.27 mm). This significant difference (*p* = 0.02) is congruent with possible antidromic activation of axons spreading through the network in the direct response case, whereas in the other condition a sufficient number of synaptic input pathways converging on a neuron need to be activated, which is most presumably bound to smaller distances.

**Figure 3:**
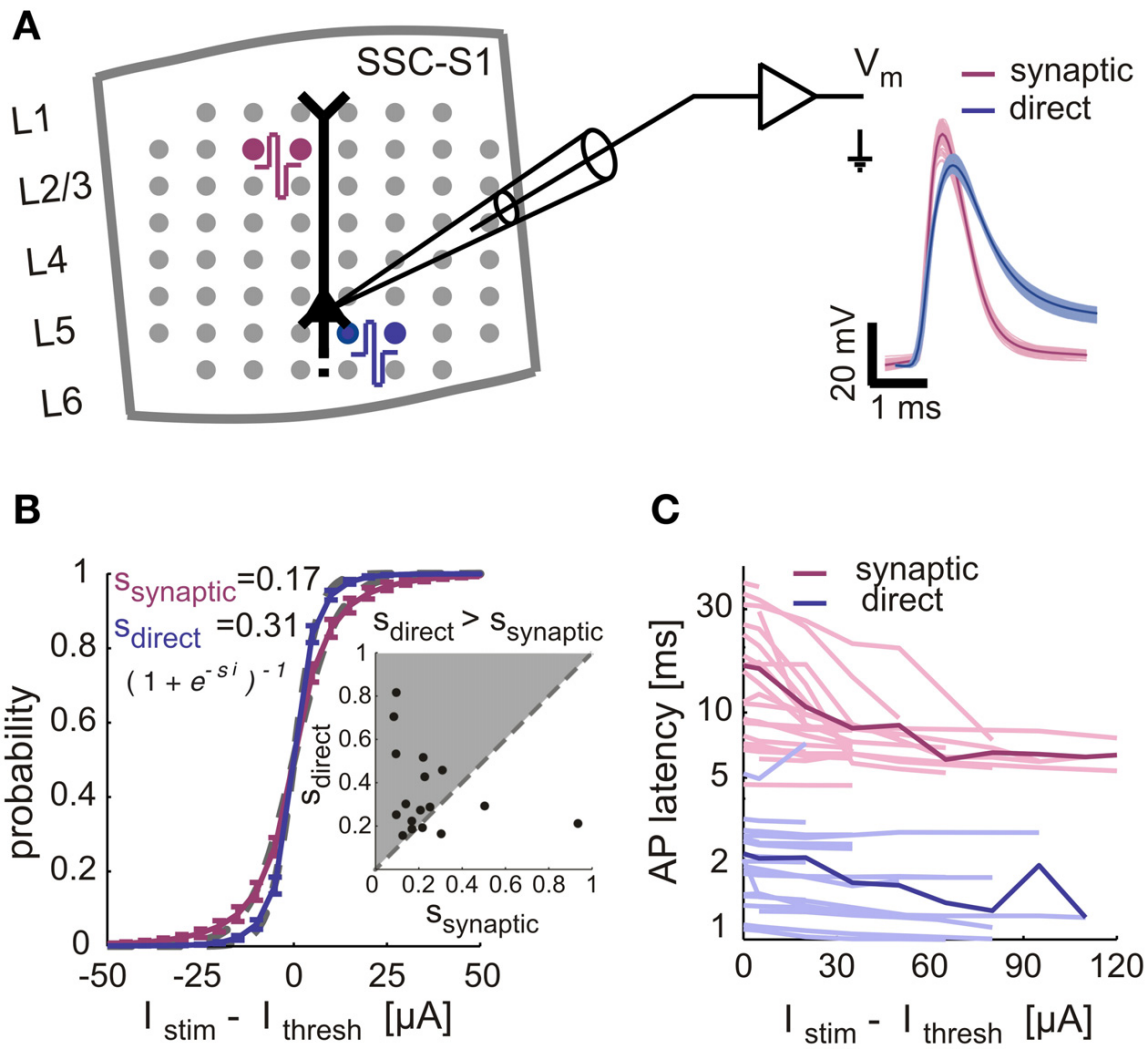
**Validation of synaptically and directly evoked neuronal responses across several and within the same neuron in cortical slices. (A)** Rat somatosensory cortical slices were coupled on MEAs for extracellular stimulation, during intracellular recordings from large L5 pyramidal cells. Red and blue circles sketch the location of bipolar stimulation sites, eliciting synaptically evoked action potentials (APs) or directly evoked APs (under additional synaptic blockade). Right panel: APs recorded in the same neuron were elicited by synaptic- (magenta) and direct stimulation (blue): waveforms are aligned to their onset, bold lines indicate mean values. **(B–C)** IO-characteristics in intracellularly recorded neurons in cortical slices. **(B)** The AP response probability, for increasing stimulus intensity, is summarized across *n* = 28 experiments performing both direct (blue, *n* = 20 neurons) and synaptic stimulation (magenta, *n* = 20 neurons) in the same neurons: gray dashed lines show the best-fit sigmoidal functions. The same fit was repeated cell-by-cell (inset) extracting the sigmoid slope parameters (i.e., ssynaptic, sdirect) and comparing individual experiments (*n* = 17 neurons) together (markers), where both stimulation types evoked responses. **(C)** The AP response latency, for increasing stimulus intensity, is plotted for the same data set as in **(B)** with thin lines representing single-cell and thick lines their average.

To further validate the extracellular-based classification of directly and synaptically evoked spikes, we have considered the intact microcircuits of cortical tissue acute slices and performed whole-cell patch-clamp recordings. While these experiments cannot support long-term (hours) measurements, they do allow a reliable comparison between direct and synaptically evoked spikes in the same neuron. By coupling brain tissue slices on MEAs, and recording from layer 5 pyramidal cells, we examined the input-output responses upon repeated extracellular stimulation, as in cultured networks. While synaptically-evoked responses were induced by stimulation of upper layers (II/III), direct responses were elicited under bath application of synaptic blockers and by stimulation near the soma, in the same cell (Figure 3A). Directly- and synaptically-evoked spikes differed in terms of width (Figure 3A, right panel), membrane potential trajectory during firing and failures (data not shown), of response mean latency (Figure 3C) and in terms of sensitivity to the stimulation intensity (Figures 3B,C). As in cultured networks, input-output responses obtained under direct stimulation were steeper than those obtained under synaptic stimulation (Figure 3B, inset, *n* = 17): across the entire data set, direct response curves were in fact 82% steeper than synaptically-evoked curves (Figure 3B). The spike mean latency and jitter were also significantly different in their input sensitivity and absolute values, with synaptically-evoked spikes occurring 5–7 times later than directly-evoked spikes (see the population summary—Figure 3C). Despite known anatomical differences (e.g., axons myelination), these results are in good qualitative agreement with the findings in cultured networks.

These experiments (Figures 2, 3) convinced us that a selection process between direct, synaptically evoked spikes may be reasonably based on the response properties close to activation threshold. Therefore, all stimulation experiments described below primarily underwent the above described stimulation and selection protocol in order to differentiate response types.

### 3.2. Impact of synaptic dynamics on long-term response fluctuations

The above results convinced us that we are capable of generating synaptically evoked spikes using extracellular stimulation delivered via substrate integrated microelectrodes. In what follows, we take advantage of this method in order to compare the statics and dynamics of directly evoked vs. synaptically mediated spikes, over extended time scales.

In the experiments described up to this point, low input frequencies (≤0.333 Hz) were employed over relatively short time scales. We now describe the impacts of a range of input frequencies on the single neuron response over extended time scales for direct (*n* = 10 neurons) and synaptically (*n* = 8 neurons) evoked spikes. Our motivation to conduct this experiment relates to previous reports showing that direct activation of a synaptically isolated neuron in frequencies higher than ~5 Hz, drives neuronal excitability to fluctuate around a threshold level, giving rise to complex intermittent responsiveness characterized by long range temporal correlations and 1/*f* statistics (e.g., Gal et al., 2010).

**Figure 4:**
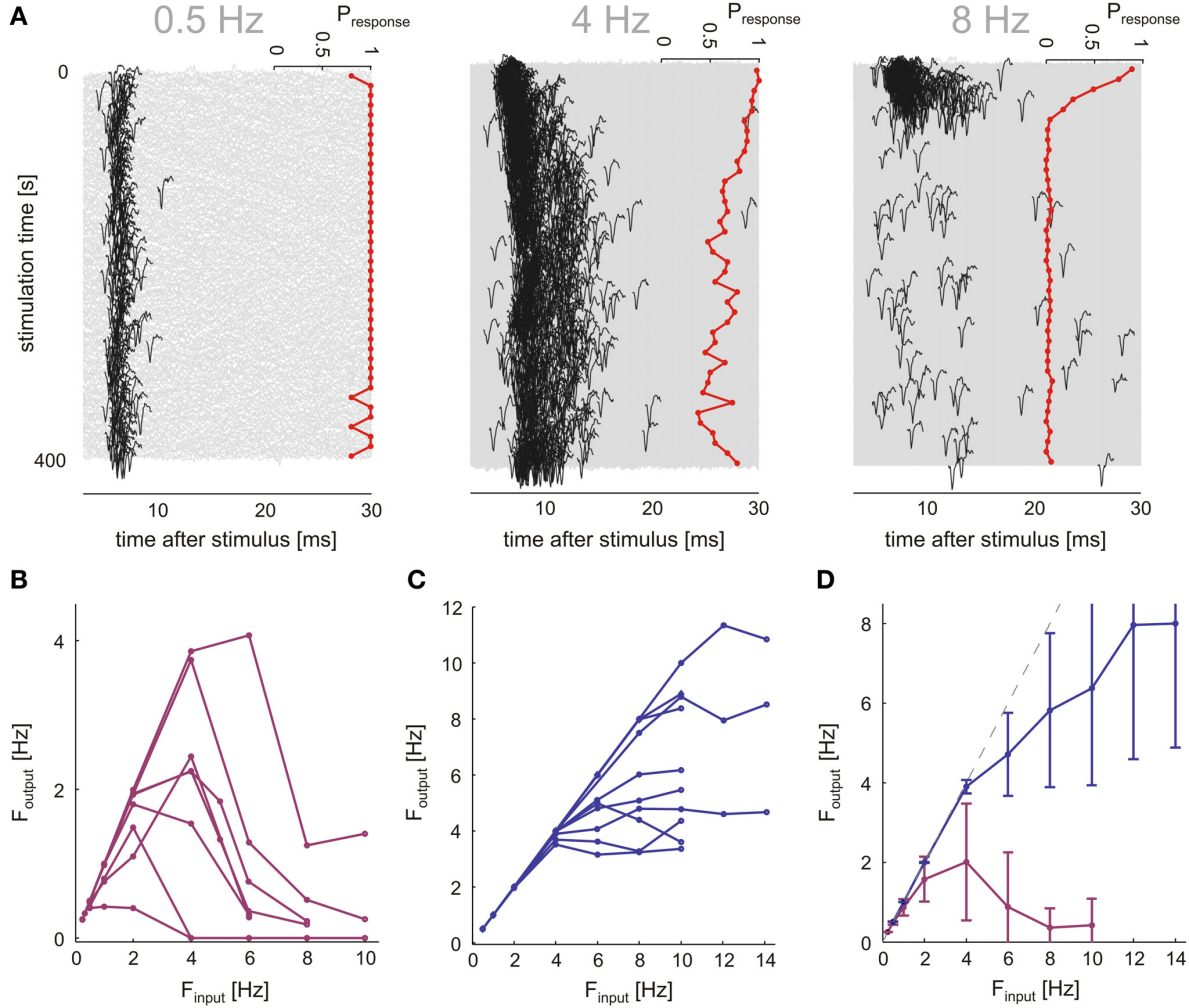
**Synaptic layer imposes low-pass filter on neuron input**. Networks (*n* = 7) were stimulated by a single electrode, responses were classified into synaptic (*n* = 8) and direct responses (*n* = 10) by the extent of delay at threshold stimulation amplitude in an experiment shown in Figure 2. **(A)** Response transients at different stimulation frequencies in a synaptically stimulated neuron are shown. All response traces within a 400 s stimulation session are plotted (gray), first spikes (black) indicated by using identical voltage scales. Binned response probabilities (bin size = 10 s), are plotted at right side of each panel. At 4 Hz, the neuron exhibits rich firing dynamics in contrast to almost steady rates at higher and lower frequencies (0.5, 8 Hz). **(B,C)** Input-Output curves display the response rate of the neuron (calculated over the last 200 s of each stimulation epoch), plotted as a function of the stimulation rate. **(D)** Mean and SD of the IO-curves shown in **(B,C)**. Direct responses only reach sporadic firing modes at input rates above 4 Hz, significantly higher frequencies than synaptic response output rate.

Neurons were stimulated with a range of frequencies (0.5, 1, 2, 4, 6, 8, 10, 12, 14 Hz), over 400 s in each frequency. Frequency sessions were applied in a random order; a 15 min break (no stimulation) separated between sessions. Each stimulation frequency was applied twice. Figure 4A shows the results of one such experiment, a case of synaptically evoked spikes: At a low stimulation frequency (0.5 Hz) the response is marked by a reliable 1:1 characteristic and stabilizes at a more or less fixed latency. At a higher stimulation frequency (middle panel of Figure 4A, 4 Hz), both the response latency and the rate of response failures gradually increase and markedly fluctuate. As the stimulation frequency is further increased (right panel of Figure 4A, 8 Hz), following a short transient phase the neuron seems to almost stop responding; after a 15 min break (inter session interval) the neuron fully recovers from this barely-responsive mode (not shown). Only neurons remaining responsive through all stimulation sessions were used. In Figure 4B, the input-output frequencies for the case of synaptically evoked spikes are plotted (8 different neurons); these plots were generated by stimulating the neurons as explained and demonstrated above. The output firing rate was calculated from the last 200 s of each session (averaged over the two repetitions). While different neurons do show different input-output frequency relations, they all have a non-monotonic shape with a maximal output rate in response to an input frequency around 2–6 Hz, a result consistent with sensitivity of synaptic resources to the activation rate. This result stands in marked contrast to the case of direct stimulation (Figure 4C), where beyond a critical stimulation frequency (typically ≥6 Hz in the present study), the output rate is practically insensitive to the input frequency, consistent with the system being sensitive to the *output* rate.

**Figure 5:**
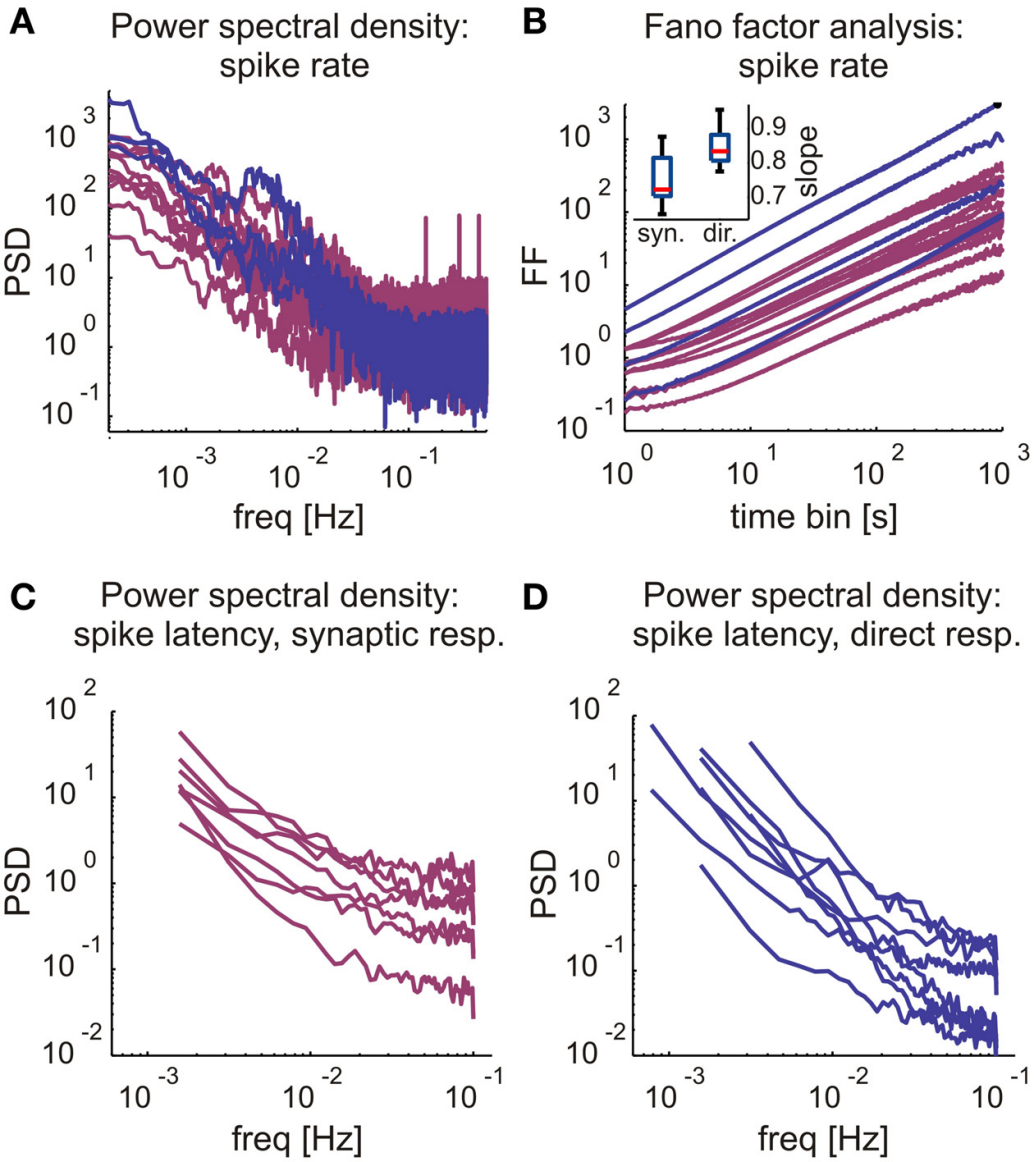
**Long-term response rate and latency dynamics in direct and synaptic responses. (A)** Firing rate periodogram, comparing synaptic (magenta) to direct (blue) response fluctuation statistics plotted on a log-log scale. **(B)** Fano Factor characteristics across various bin sizes, comparing synaptic (magenta) to direct (blue) response fluctuation statistics plotted on a log-log scale. Inset: Boxplot of the estimated slopes (α_*D*_) of the Fano factor curves. Note that the scaling exponents of the Fano Factor do slightly differ in the two stimulation regimes, in directly evoked spikes they are higher (0.85 ± 0.062, mean ± *SD*; *n* = 9) compared to those estimated from synaptically evoked activity (0.755 ± 0.074, mean ± *SD*; *n* = 10). Boxplot: horizontal red line, median; blue box, 25th–75th percentile, red cross, outlier. In **(A,B)**, analyzes were done according to Gal et al. (2010) made on direct responses. Therefore, focusing on synaptic response statistics, only four direct response rate statistics were plotted as a reference, yet all were included in slope comparisons. **(C,D)** Power spectral densities of the latency values of synaptic (left) and direct (right) responses in modes of sporadic firing. PSD calculated with Welch's method (bin size = 5 s). Neurons showing global trends in spike rate, latency and amplitude were excluded, as well as neurons with a significant probability (> 5%) of empty bins. Responses were classified to synaptic (*n* = 7) and direct responses (*n* = 8) as described above.

A comparison of the averaged stimulus-response curves (Figure 4D) reveals that the rate at which a neuron may be activated via synaptic transmission is low compared to the range of activation rates that drive neurons to intermittency due to excitability dynamics as described in previous works. Hence, it is possible that rate fluctuations of synaptically evoked spikes, obtained at relatively low stimulation frequency (as demonstrated in the 400 s stimulation session of Figure 4A), is primarily due to dynamics in synaptic transmission rather than membrane excitability. This interpretation is further supported by analyses of synaptically evoked spikes generated in response to long (≥5 h, 6 networks) series of stimuli at a constant frequency value, at or near the frequency that maximizes the output of each given neuron (*n* = 10). The first 30 min of responses to these long stimulation series were discarded in order to dismiss transients. We find that, indeed, within the synaptic input frequency range that maximized output rate, neurons do show complex dynamics, as indicated by the power law like tail in the low frequency domain presented in Figure 5A, and the Fano factor scaling relations of Figure 5B. For comparison, results obtain by similar analyses on directly evoked spikes (in response to higher frequencies, ≥5 Hz) are shown (blue traces). The complexity of the output time series is also reflected in the fluctuations of spike latencies (Figures 5C,D).

**Figure 6:**
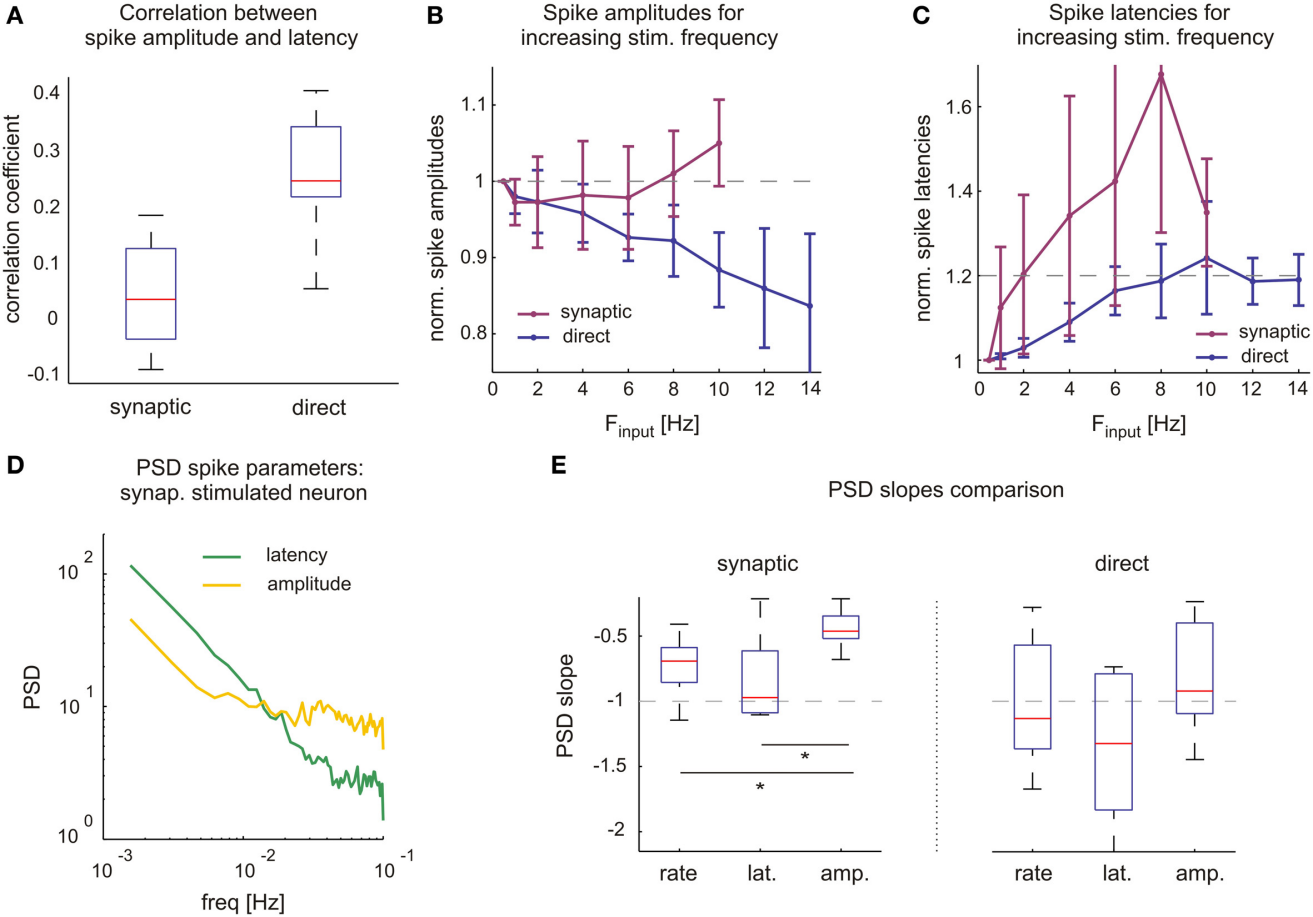
**Synaptic processes are key determinants of rich response dynamics. (A)** Unlike in synaptic mediated responses, direct response spike amplitudes and latencies are correlated. Spike parameters used from high frequency stimulation experiments shown in **(C,D)** (Figure 5). Box plot (horizontal red line, median; blue box, 25th–75th percentile). **(B)** In contrast to synaptic, the direct mediated spike amplitudes decrease significantly with increasing stimulation frequencies. Means and SDs of spike amplitudes belonging to the same neurons, shown in **(B,C)**, (Figure 4) and therefore contain different counts of data points for each stimulation frequency. In order to compare across several neurons, averaged amplitudes of each neuron are normalized by its average at 0.5 Hz. **(C)** Direct mediated spike latencies approach to and fluctuate around 120% of their initial values, while synaptic mediated response latencies increase with stimulation frequencies. Neurons and analysis like for spike amplitudes, middle panel. **(D)** Comparing power spectral density of spike latencies (green) and amplitudes (yellow) of an individual neuron stimulated synaptically. Values were normalized by SD to omit possible offsets. **(E)** Estimated PSD slopes of long-term stimulation experiment values. PSD slopes of direct response parameters vary close to −1, indicating 1/*f* characteristics. For synaptic mediated responses the same is true in spike rate and latency, however PSD slopes of spike amplitude (−0.45 ± 0.15, mean ± *SD*) differ significantly (*t*-test with *p*-value < 0.02 comparing to both latency and rate). Slope estimates were performed with linear regression of logarithm of the data.

We see that while rate and latency dynamics exhibited in direct responses can be exclusively attributed to fluctuations of membrane excitability, interpretations are more subtle when neurons are stimulated synaptically, as both synaptic transmission and neuronal excitability might contribute to response variability. To expose their relative contributions, excitability fluctuations in synaptic mediated responses over extended time scales must be monitored. To this aim we took advantage of the fact that variations in neuronal excitability are reflected in spike amplitude and its fluctuations. Note that, unlike spike latency, spike amplitude in our experimental settings is independent of input strength (e.g., Figure 2 as well as in our intracellular experiments, not shown). Indeed, in agreement with previous reports describing direct responses, Figure 6A shows that spike amplitudes and latencies are correlated and thereby follow Hodgkin-Huxley predictions on neuronal excitability. However, Figure 6A also shows that, when stimulated synaptically, spike amplitudes and latencies were not correlated, suggesting that in this case the latency fluctuations reflect different underlying processes. We have used these observations in our analyses of the relative contributions of synaptic and excitability dynamics, by monitoring spike amplitudes in neurons stimulated with different frequencies (Figure 6B, the same neurons as shown in Figure 4D). While directly evoked response amplitudes decrease with increasing stimulation frequencies and response failure rates, a different picture emerges in synaptically evoked responses. Here, spike amplitudes remain relatively unchanged or even slightly increase with stimulation frequency; the latter can be explained by their decreasing output firing rates. We similarly analyzed the response spike latencies of the same neurons (Figure 6C). Directly evoked spike latencies approach slightly increased values (approx. 120%) and fluctuate around these, independent on stimulation frequency. In contrast, synaptic mediated response latencies seem to be highly dependent on stimulation frequencies.

The differential sensitivity of spike amplitudes to direct vs. synaptic stimulation rates, may be used as a tool to further estimate the contribution of synaptic transmission dynamics as a source of long-memory processes and complex statistics of spike rates. Figure 6D depicts the temporal statistics of spike amplitude and latency in terms of Power Spectral Density for one synaptically activated neuron. Figure 6E summarized results from many neurons, in both synaptically and directly evoked cases. In the case of synaptically evoked spikes, the slope of the amplitude spectrum is significantly lower compared to rate and latency. In direct responses, albeit wider distribution of the data, there seems to be no significant difference between the different spectra; all are close to unity.

## 4. Discussion

The responsiveness of network embedded spiking neurons *in vivo* and *in vitro* varies significantly over time (Carandini, 2004; Mazzoni et al., 2007). The hallmarks of this variability are broadly distributed (often scale free) statistics, reflected in long-range correlations, quasi-stable response patterns and, more generally, long-memory processes that extend to behaviorally relevant scales (Lowen and Teich, 1996). Combined with well-documented long-memory processes in measures of large-scale network activity *in vivo* and *in vitro* (Segev et al., 2002; Beggs and Plenz, 2003), the complexity of single neuron response variability is likely to reflect the complex dynamical features of the surrounding networks (Arieli et al., 1996; Azouz and Gray, 1999). But experimental observations and theoretical considerations suggest that even single neurons, in isolation from their surrounding networks, exhibit long-memory processes, including 1/*f* statistics, quasi-stable response patterns and long-range correlations (Lowen et al., 1999; Marom, 2009). These cell-intrinsic dynamics are interpreted as consequences of threshold fluctuations resulting from kinetics at the ion channel level and other subcellular processes (e.g., Fleidervish et al., 1996; reviewed in Marom, 2010).

Our study focuses on the intermediate level, between the network and the single neuron, examining those subsets of synapses eliciting an action potential when activated simultaneously. In particular we examine a subset of synapses that constitutes a stimulus specific entity, and—for the purposes of the present study—we assume it to be composed of a relatively stable population of synapses per given sensory input (Jia et al., 2010; Chen et al., 2013). The experiments described in the present study were specifically designed to estimate the contribution of a stimulus specific synaptic population to long-term single neuron response fluctuations.

By employing a method that is tailored for controlled long-term activation of a single neuron through a synaptic population, we show that within the range of physiological activation frequencies, long memory in neuronal response spike time series is significantly impacted by synaptic dynamics. This conclusion is supported by the following observations: (1) The stimulation rate required in order to obtain long memory and complex statistics in neuronal output, when the neuron is activated synaptically (over periods of minutes), may be as low as 2 Hz. Such low stimulation rates evoke 1:1 responsiveness in directly stimulated neurons (Figure 4D), and thereby cannot give rise to long-term threshold fluctuations. Moreover, the slopes in Figure 4B are monotonically decreasing already above ca. 1 Hz; the summary of Figure 4D suggests that in any event, a neuron cannot be activated for long periods of time by a given stimulus specific synaptic ensemble in a rate that is sufficient to evoke excitability-mediated complex response statistics. Note that our method, entailed by the need for long-term stable recordings of synaptic mediated responses, is *a priori* limited in the sense of synaptic mediated responses unavoidably involve membrane excitability effects in the vicinity of the synapses studied. In this context, the above reported difference in sensitivity to input frequencies enables dissecting and interpreting the effects of synaptic vs. excitability processes on neuronal response dynamics. (2) Spike amplitude—an indirect measure of neuronal excitability (Henze and Buzsaki, 2001; de Polavieja et al., 2005 etc.)—does not decrease with increasing stimulation frequencies (raising failure rates) in synaptically mediated spikes. This is in contrast to directly evoked responses (Figure 6A). (3) In contrast to direct responses, synaptic mediated response spike amplitude and latency fluctuations are un-correlated, displaying different long-term statistics (Figures 6B–D).

Single neuron response variability may be (and probably is) affected by processes at several organization levels, from membrane excitability machinery to synaptic, and network dynamics (Azouz and Gray, 1999; Harsch and Robinson, 2000; Desai et al., 2002). These sources seem to occupy different, although partially overlapping, ranges of activation rates. For a membrane excitability machinery to express long-term complex statistics, relatively high input rates (above ca. 5 Hz) are required. Synaptically originated long-term complex spike time series emerge at slightly lower input frequencies (as low as 2 Hz). Network complexity is exposed at an even lower frequency range (~0.1 Hz) (Beggs and Plenz, 2003; Eytan and Marom, 2006; Pasquale et al., 2008).

Our interpretation of synaptic dominance, in the emergence of complexity and long-memory processes in synaptically driven neurons, is congruent with published reports that demonstrate synapse filtering properties and response variability over shorter time scales (Thomson, 1997; Tsodyks and Markram, 1997; Markram et al., 1998; Fortune and Rose, 2001; Chung et al., 2002). However, it is important to acknowledge that the synaptic dominance demonstrated in the experiments described here, holds for the case of a single activation pathway to the neuron reactivating a distinct subset of synapses. It is likely that a neuron can be activated via several such paths (representing for example different network states or sensory pathways), resulting in higher activation rates of the membrane. Depending on the overlap of those synaptic ensembles, temporal complexity can rise through synaptic processes and as well in the machinery of excitability (Gal et al., 2010).

## Author contributions

Sebastian Reinartz, Conception and design of the experiments in cortical cultures; acquisition and analysis of cortical culture data; interpretation of cortical culture and cortical slice data; writing and revision of manuscript draft and other required contributions. Istvan Biro, Design of cortical slice experiments; acquisition and analysis of cortical slice data; interpretation of cortical slice data and other required contributions. Asaf Gal, Interpretation of cortical culture data; revision of manuscript draft and other required contributions. Michele Giugliano, Conception and design of the experiments in cortical slices; interpretation of cortical slice data; writing and revision of manuscript draft and other required contributions. Shimon Marom, Conception of the experiments in cortical cultures; interpretation of cortical culture and cortical slice data; revision of manuscript draft and other required contributions.

## Funding

The research leading to these results has received funding from the European Union's—Seventh Framework Programme [*FP7*/2007−2013] under grant agreement #FP7-269459 Coronet, and was supported by a grant from the Ministry of Science and Technology, Israel and funding organization of EU countries. Funding from the Univ. Antwerp (NOI-BOF2009), the IWT & EC-FP7 MATERA+ (grant n. 90455), Belgian Science Policy Office (BELSPO) and IAP-VII programme is kindly acknowledged.

### Conflict of interest statement

The authors declare that the research was conducted in the absence of any commercial or financial relationships that could be construed as a potential conflict of interest.
